# Latent profile analysis of AI anxiety among medical imaging students: a cross-sectional study based on emotional flexibility and future employment anxiety

**DOI:** 10.3389/fpsyg.2026.1882645

**Published:** 2026-10-20

**Authors:** Lixing Lei, Ling Hu, Min Zhang, Xin He, Liu Qianqian, Lingling Tang, Nian Liu, Xiao-hua Huang

**Affiliations:** 1 Department of Radiology, Affiliated Hospital of North Sichuan Medical College, Nanchong, China; 2 School of Medical Imaging, North Sichuan Medical College, Nanchong, China

**Keywords:** AI anxiety, emotional flexibility, future employment anxiety, latent profile analysis, medical imaging students

## Abstract

**Objective:**

This study aimed to identify latent profiles of AI anxiety among medical imaging students and to examine the associations of emotional flexibility, future employment anxiety, and demographic variables with profile membership, thereby providing empirical evidence for differentiated psychological intervention strategies.

**Methods:**

A cross-sectional survey was conducted using convenience sampling among 488 medical imaging students recruited from North Sichuan Medical College in Sichuan Province, China. Data were collected using the AI Anxiety Scale, the Emotional Flexibility Scale, the Future Employment Anxiety Scale, and a demographic questionnaire. Latent profile analysis was used to identify AI anxiety profiles, and the three-step BCH and R3STEP procedures were used to examine distal outcomes and covariates while accounting for classification uncertainty.

**Results:**

Latent profile analysis identified three severity-ordered AI anxiety profiles: a Medium AI anxiety profile (65.78%), a High AI anxiety profile (22.75%), and a Low AI anxiety profile (11.48%). The High profile scored most prominently on Job Replacement and Sociotechnical Blindness anxiety. Emotional flexibility showed a nonlinear distribution across profiles: highest in the Low profile (BCH *M* = 3.975), lowest in the Medium profile (3.282), and intermediate in the High profile (3.805). Future employment anxiety increased monotonically across the profiles (Low: 2.285; Medium: 3.255; High: 4.126; all pairwise *p* < 0.001) and independently distinguished all profiles in the classification-error-adjusted (R3STEP) analysis. Emotional flexibility was lowest in the Medium profile and did not differ between the High and Low profiles (*p* = 0.271); in the classification-error-adjusted analysis, it distinguished the High from the Medium profile but not the Low from the Medium profile.

**Conclusion:**

The findings support differentiated psychological support tailored to distinct AI anxiety profiles, and suggest that AI literacy education and career planning guidance should be systematically integrated into medical imaging curricula.

## 1 Introduction

The penetration of artificial intelligence (AI) technology into the healthcare sector has exhibited a marked growth trajectory, particularly within the field of medical imaging (Alexander et al., 2020; Gou et al., 2024). The application of deep learning algorithms in diagnostic imaging now encompasses a broad range of domains, including pulmonary nodule detection, breast cancer screening, retinal disease identification, and fracture classification (Yoo et al., 2020; Nam et al., 2019; Kim et al., 2023). Previous research has demonstrated that the diagnostic accuracy of deep learning algorithms in specific tasks has approached or even surpassed that of practicing radiologists (Chartrand et al., 2017; Rajpurkar et al., 2018). More importantly, AI technology holds substantial application potential in technical domains such as image post-processing, image reconstruction, and quality control (Willemink and Noël, 2019; Hellwig et al., 2023). Consequently, students specializing in both diagnostic radiography and medical imaging technology face mounting pressure from the prospect of their professional roles being redefined or partially supplanted. As the future core practitioners in this field, medical imaging students' psychological perceptions of and emotional responses to AI technology bear implications not only for individual mental health and academic adjustment but also, at a deeper level, for the transformation of talent cultivation models and the sustainable development of the clinical workforce (Zhai et al., 2025; Yan et al., 2025). Accordingly, the present study undertakes a systematic investigation of the psychological stress responses of medical imaging students in the face of AI technology, with particular emphasis on their anxiety experiences.

AI anxiety refers to a diffuse emotional experience of worry, unease, and fear that arises in individuals when confronted with the development, application, and potential societal implications of AI technology (Li et al., 2020; Frenkenberg and Hochman, 2025). According to the technology threat perception theory (Liang and Xue, 2009), cognitive appraisal of AI technology plays a central role in the generation of anxiety, encompassing multiple dimensions such as fear of job displacement, discomfort with human–machine interaction, and doubt regarding one's own capacity for technological adaptation (Wang G. et al., 2025; Fang et al., 2026). Previous research has revealed significant associations between AI anxiety among college students and academic burnout, career decision-making difficulties, and technology avoidance behaviors (Wang and Gao, 2025; Eren et al., 2025; Li et al., 2025). However, the existing literature has predominantly treated AI anxiety as a homogeneous continuous variable, overlooking qualitative differences that may exist in patterns of AI anxiety experiences across individuals. Notably, research specifically examining AI anxiety among medical imaging students—a population whose professional identity is highly intertwined with AI technology—remains exceedingly scarce. Given the distinctive characteristics of their discipline, this population may exhibit unique anxiety features and differentiation patterns that diverge from those of the general college student population.

Conventional variable-centered approaches assume sample homogeneity on the target construct and characterize overall trends through group means or the exploration of linear relationships among variables (Muthén and Muthén, 2000); however, such methods are inherently limited in their capacity to uncover heterogeneous subgroup structures latent within the population. Latent profile analysis (LPA) is capable of identifying distinct latent classes or profiles characterized by different combinations of features based on individuals' response patterns across multiple observed indicators (Hickendorff et al., 2018; Yang et al., 2025). In previous research, LPA has been widely employed to identify distinct anxiety profile types (Liu et al., 2022; Ding and Zhang, 2025), and different profiles have frequently exhibited significant differences in both antecedent and outcome variables. Nevertheless, no study to date has applied LPA to explore and identify latent classes of AI anxiety among medical imaging students. On this basis, the present study employs LPA to examine, from a multidimensional perspective of AI anxiety, whether distinct, severity-ordered AI anxiety subgroups exist among medical imaging students, and further investigates the predictive roles of demographic factors, emotional flexibility, and future employment anxiety in profile membership, with the aim of deepening the understanding of the internal structure of AI anxiety from the perspective of individual differences.

Emotional flexibility refers to the capacity to adaptively regulate, shift among, and deploy different emotional response strategies in accordance with situational demands, reflecting the adaptive functioning of the emotional system (Kobylińska and Kusev, 2019; Sanchez-Lopez, 2021). According to acceptance and commitment therapy theory (Hayes et al., 2006), rather than rigidly avoiding or suppressing negative emotional experiences, individuals are encouraged to perceive emotions with an open and accepting attitude and to make behavioral choices guided by personal values (Kashdan et al., 2006; Li et al., 2026). Specifically, individuals with high emotional flexibility are better equipped to engage in cognitive reappraisal and emotion regulation when confronted with uncertain situations and threatening events, thereby reducing both the probability and intensity of negative emotions such as anxiety and depression (Wang X. et al., 2025; Sharma and Singh, 2025). In the context of AI-driven transformation, students' emotional flexibility may predispose them to perceive AI advancement as an adaptable challenge rather than an existential threat, thereby buffering the emergence of AI anxiety (Çavu ş Alan and Karabag Aydin, 2026). Regrettably, very few studies have explored the relationship between emotional flexibility and latent profiles of AI anxiety. Accordingly, the present study hypothesizes that significant differences in emotional flexibility exist across different AI anxiety profiles. Individuals with higher emotional flexibility are more likely to belong to a low AI anxiety profile, whereas those with lower emotional flexibility are more likely to exhibit a multi dimensionally pronounced AI anxiety pattern.

Future employment anxiety refers to the sustained worry and unease experienced by individuals regarding their career prospects, employability, and potential for professional development (Wang C. et al., 2025). The impact of AI technology on traditional imaging positions further intensifies future employment anxiety (Shen and Zhou, 2024). Specifically, students may fear that AI will substantially reduce the demand for diagnostic imaging and technical positions, or worry that their existing knowledge and skill sets will be inadequate to meet the occupational requirements of the AI era. Drawing on the cognitive–emotional spillover (Zillmann, 1971), individuals' anxiety perceptions regarding AI technology may further exacerbate concerns about future employment by activating negative cognitions about career prospects (Kim and Lee, 2025). A limited body of existing research has provided preliminary evidence for a positive association between AI-related concerns and perceived career uncertainty (Dag et al., 2026); however, no study has yet examined differences in future employment anxiety levels among students belonging to different AI anxiety classes from a latent profile perspective. Therefore, the present study posits that profiles characterized by higher levels or more complex patterns of AI anxiety will be associated with more intense future employment anxiety experienced by their members.

Based on the aforementioned research gaps, the present study aims to achieve the following objectives:

(1) to identify the number and characteristics of latent AI anxiety classes among medical imaging students using latent profile analysis;
(2) to examine the predictive effects of demographic and academic variables on profile membership;
(3) to investigate differences in emotional flexibility across distinct AI anxiety profiles;
(4) to test whether different AI anxiety profiles exhibit differential levels of future employment anxiety.

Through a person-centered analytical strategy and a typological perspective, this study endeavors to enrich the understanding of AI anxiety among medical imaging students and to provide empirical support for the development of targeted psychological intervention programs and educational reform strategies.

## 2 Methods

This study employed a cross-sectional survey design to analyze the latent class structure of AI anxiety among medical imaging students and its associated factors. The research protocol was reviewed and approved by the Medical Ethics Committee of the Affiliated Hospital of North Sichuan Medical College prior to data collection (No.: 2025ER447-1). Electronic informed consent was obtained from all participants.

### 2.1 Participant recruitment

Between March to April 2026, full-time undergraduate students enrolled in medical imaging–related programs were recruited from North Sichuan Medical College in Sichuan Province, China, using convenience sampling. The research team first contacted and obtained cooperation approval from the student affairs offices of the respective schools of medical imaging. An electronic questionnaire was developed using a professional data collection platform (Jian Shu) and distributed as a self-administered online survey to the target population. The introductory page of the questionnaire provided a detailed description of the study background along with assurances of anonymity and confidentiality. Participants were permitted to proceed to the formal survey only after confirming their informed consent.

The inclusion criteria were as follows: (1) full-time undergraduate students enrolled in diagnostic medical imaging or medical imaging technology programs; (2) students spanning all academic years from the first through the fifth year; (3) ability to comprehend the questionnaire content and complete it independently; and (4) voluntary participation in the study with provision of informed consent.

The exclusion criteria were as follows: (1) students not in active academic standing during the survey period due to suspension of studies or leave of absence; (2) individuals who self-reported a clinically diagnosed severe psychiatric disorder in an acute episode; and (3) respondents whose submissions were deemed invalid due to excessively short completion times or overtly patterned response styles.

Regarding sample size, Monte Carlo simulation studies by Tein et al. (2013) indicate that the statistical power to detect the correct number of latent classes in latent profile analysis depends jointly on sample size, the number of indicators, and the degree of separation between classes, rather than on a single universal threshold. For adequately separated classes, adequate power has been reported with samples of a few hundred participants, whereas less well-separated classes may require substantially larger samples. The present sample (*N* = 488) is therefore expected to provide sufficient power to recover two- to four-class solutions under the conditions most relevant to this study.

During the data collection phase, a total of 561 students participated in the online survey. During the data cleaning process, incomplete questionnaires (*n* = 26), responses with abnormally short completion times (*n* = 19), and responses exhibiting overtly patterned answering styles (*n* = 28) were excluded, yielding a final analytic sample of 488 participants (effective response rate = 86.99%). Among these, 233 were male (47.7%) and 255 were female (52.3%). Detailed demographic information is presented in Table 1.

**Table 1:** Summary of demographic characteristics of medical imaging students.

Variables	Items	Number (*N*)	Proportion (%)
Gender	Male	233	47.7%
	Female	255	52.3%
Grade	Freshman year	184	37.7%
	Sophomore Year	131	26.8%
	Junior Year	96	19.7%
	Senior year	37	7.6%
	Fifth year of university	40	8.2%
Educational system	Four-year program	229	46.9%
	Five-year program	259	53.1%
Major	Medical imaging technology	195	40.0%
	Medical imaging diagnosis	293	60.0%
Only child	No.	267	54.7%
	Yes.	221	45.3%
Internship or internship experience	No.	294	60.2%
	Yes.	194	39.8%
Frequency of AI use in the past week	1~2 times	223	45.7%
	3~5 times	144	29.5%
	6 times and above	103	21.1%
	Unused	18	3.7%
Age		20.92 ± 2.838	

### 2.2 Instruments tools

#### 2.2.1 AI anxiety scale

The Chinese version of the AI Anxiety Scale, developed by Wang and Wang (2022), comprises 21 items organized into four dimensions: Learning, Job Replacement, Sociotechnical Blindness, and AI Configuration. This scale was employed in the present study to measure AI anxiety among medical imaging students, with responses recorded on a 5-point Likert scale ranging from 1 (strongly disagree) to 5 (strongly agree). A sample item is “Learning to understand all the special functions related to AI technology/products makes me feel anxious.” Total scores range from 21 to 105, with higher scores indicating greater levels of AI anxiety. In the present study, Cronbach's alpha coefficient for the AI Anxiety Scale was 0.938, indicating excellent internal consistency.

#### 2.2.2 Emotional flexibility scale

The Chinese version of the Emotional Flexibility Scale, developed by Fu et al. (2018), comprises 10 items encompassing three dimensions: positive emotion regulation, negative emotion regulation, and emotional communication. A sample item is “When I have positive emotions, if the people around me are all busy, I will not express my feelings.” This scale was used in the present study to assess emotional flexibility among medical imaging students, with responses rated on a 5-point Likert scale ranging from 1 (never true) to 5 (always true). Total scores range from 10 to 50, with higher scores reflecting greater emotional flexibility. In the present study, Cronbach's alpha coefficient for the Emotional Flexibility Scale was 0.873, demonstrating good internal consistency.

#### 2.2.3 Future employment anxiety scale

The Chinese version of the Future Employment Anxiety Scale (FEAS), developed by Wang X. et al. (2025), comprises 17 items organized into four dimensions: personal competence, knowledge application, career replaceability, and social relationships. A sample item is “I worry that I will not be able to manage relationships with colleagues well in my future job.” This scale was employed in the present study to measure future employment anxiety among medical imaging students, with responses recorded on a 5-point Likert scale ranging from 1 (strongly disagree) to 5 (strongly agree). Total scores range from 17 to 85, with higher scores indicating more pronounced future employment anxiety. In the present study, Cronbach's alpha coefficient for the Future Employment Anxiety Scale was 0.932, indicating good internal consistency.

### 2.3 Statistical analysis

All analyses were performed using Mplus 8.3 and SPSS 27.0 with α = 0.05. Preliminary analyses included reliability testing, descriptive statistics, correlation analysis, and common method bias testing. Latent profile analysis (LPA) was conducted using Mplus 8.3 with maximum likelihood estimation with robust standard errors (MLR). The four AI Anxiety Scale dimension scores served as continuous indicators, and models with one to five latent profiles were fitted sequentially. A class-invariant diagonal covariance structure was specified (variances freely estimated and constrained equal across classes; within-class covariances fixed to zero, consistent with the local-independence assumption of LPA). All models used 200 initial-stage random starts and 50 final-stage optimizations; the best log-likelihood was verified to be replicated across random starts, and all models terminated normally. Model selection considered the Akaike Information Criterion (AIC), Bayesian Information Criterion (BIC), sample-size-adjusted BIC (aBIC), the Lo–Mendell–Rubin adjusted likelihood ratio test (LMR-LRT), the parametric bootstrap likelihood ratio test (BLRT), entropy (as an index of classification precision), class proportions (minimum ≥5%), and theoretical interpretability.

To account for classification uncertainty, secondary analyses used the three-step procedures implemented in Mplus 8.3. Emotional flexibility and future employment anxiety were treated as distal outcomes and analyzed with the BCH method to test profile differences in these correlates; separately, to identify which correlates and demographic covariates most strongly and independently characterized profile membership, emotional flexibility, future employment anxiety, and only-child status were entered simultaneously as covariates in an R3STEP multinomial regression with the Medium profile as the reference category. No variable was treated as both outcome and covariate within a single model. Given heterogeneous variances (Levene's test, *p* < 0.001 for both outcomes), Welch's ANOVA and Games–Howell *post hoc* comparisons were used for group contrasts, with Cohen's d reported as the effect size. Variance inflation factors (VIFs) were computed for the predictor variables to assess multicollinearity. Because of the conceptual overlap between the Job Replacement dimension of the AI Anxiety Scale and the Career Replaceability dimension of the Future Employment Anxiety Scale, a two-factor confirmatory factor analysis was used to assess discriminant validity (average variance extracted and the Fornell–Larcker criterion), and a targeted overlap check was conducted in which the four Career Replaceability items were excluded and the analyses re-estimated.

## 3 Results

### 3.1 Common method bias testing and descriptive statistics

Given that this study relied on self-report questionnaires for data collection, with all research variables derived from the subjective evaluations of the same respondents, and considering the potential influence of social desirability, common method bias may be a concern. To mitigate this risk, procedural remedies were implemented during questionnaire design, including randomizing or alternating the presentation order of items from different scales, incorporating reverse-scored items, and ensuring the anonymity and confidentiality of responses.

Harman's single-factor test was employed to assess common method bias. All measurement items were entered into an unrotated exploratory factor analysis. The results revealed five factors with eigenvalues greater than 1, with the first factor accounting for 32.508% of the total variance, which fell below the critical threshold of 40%. These findings indicate that common method bias was not a serious concern in the present study.

The skewness values of the core study variables ranged from −0.435 to 0.535, and the kurtosis values ranged from 0.142 to 0.966. All values satisfied the criteria of skewness <|2| and kurtosis <|7|, thereby meeting the assumption of approximate normality, as shown in Table 2.

**Table 2:** Descriptive statistics of core study variables.

Variables	*M*	SD	Skewness	Kurtosis
AI anxiety	3.114	0.661	−0.435	0.966
Emotional flexibility	3.486	0.612	0.535	0.142
Future employment anxiety	3.352	0.701	−0.343	0.583

*M* = Mean; *SD* = standard deviation.

### 3.2 Discriminant validity

A two-factor confirmatory factor analysis (AI anxiety, 21 items; future employment anxiety, 17 items) fitted the data substantially better than a one-factor model (Δχ^2^(1) = 791.85, *p* < 0.001; two-factor: χ^2^(664) = 2571.51, RMSEA = 0.077, CFI = 0.819, SRMR = 0.065; one-factor: χ^2^(665) = 3363.36, RMSEA = 0.091, CFI = 0.744, SRMR = 0.077), indicating that the two instruments are not fully redundant. However, the latent correlation between the two constructs was r = 0.792 (*r*^2^ = 0.627), and the average variance extracted for each construct (AI anxiety AVE = 0.418; future employment anxiety AVE = 0.471) was lower than this squared latent correlation, so that the Fornell–Larcker criterion for discriminant validity was not met. This indicates substantial shared variance between the two constructs, consistent with their substantive adjacency (particularly Job Replacement ↔ Career Replaceability), which is acknowledged as a limitation.

The correlation matrix of the core variables is reported in Table 3. AI anxiety total correlated strongly with future employment anxiety total (*r* = 0.741, *p* < 0.001). The AI Job Replacement dimension correlated with the FEAS Career Replaceability dimension (*r* = 0.696) and remained strongly associated with future employment anxiety even after excluding the Career Replaceability items (*r* = 0.718). Emotional flexibility was not significantly correlated with AI anxiety (*r* = −0.032) or with future employment anxiety (*r* = −0.022).

**Table 3:** Correlation analysis of core study variables.

Variables	1	2	3	4	5	6	7
1. AI anxiety	1						
2. Emotional flexibility	−0.032	1					
3. Future employment anxiety	0.741^***^	−0.022	1				
4. FEAS excluding Career Replaceability	0.738^***^	−0.030	0.991^***^	1			
5. FEAS Career Replaceability	0.678^***^	0.001	0.929^***^	0.869^***^	1		
6. AI Job Replacement	0.881^***^	−0.004	0.731^***^	0.718^***^	0.696^***^	1	
7. AI Sociotechnical Blindness	0.831^***^	−0.004	0.695^***^	0.687^***^	0.647^***^	0.772^***^	1

^***^p < 0.001.

### 3.3 Latent profile analysis of AI anxiety

To further explore the latent profile structure of AI anxiety among medical imaging students, the present study used the four dimension scores of the AI Anxiety Scale as continuous indicator variables and sequentially fitted models containing one through five latent profiles. The fit indices for each model are summarized in Table 4.

**Table 4:** The fit indices for each model are summarized.

Profile	AIC	BIC	aBIC	Entropy	LMR (*p*)	BLRT (*p*)	Proportion
1	4676.117	4709.639	4684.247	–	–	–	–
2	4184.794	4239.268	4198.007	0.899	<0.001	<0.001	16.19%/83.81%
3	3862.850	3938.276	3881.144	0.850	0.001	<0.001	64.60%/23.92%/11.48%
4	3778.010	3874.387	3801.386	0.812	0.010	<0.001	9.43%/42.42%/36.89%/11.27%
5	3723.056	3840.384	3751.513	0.832	0.503	<0.001	5.12%/8.19%/34.84%/41.19%/10.66%

AIC, Akaike Information Criterion; BIC, Bayesian Information Criterion; aBIC, Adjusted BIC; LMR, Lo-Mendell-Rubin Likelihood Ratio Test; BLRT, Bootstrap Likelihood Ratio Test.

As the number of latent classes increased, the AIC, BIC, and aBIC information criteria all exhibited a consistent downward trend, indicating progressive improvement in model fit. Regarding likelihood ratio tests, the LMR-LRT was significant for the 2- vs. 1-class (*p* < 0.001), 3- vs. 2-class (*p* = 0.001), and 4- vs. 3-class (*p* = 0.010) comparisons, but not for the 5- vs. 4-class comparison (*p* = 0.503), indicating that the 5-profile model did not provide a significant improvement in fit over the 4-profile model. The information criteria (AIC, BIC, aBIC) and the BLRT therefore favored the four-profile solution. Nevertheless, the three-profile solution was retained as the primary model on substantive grounds: parsimony; higher classification precision (entropy = 0.850 vs. 0.812 for the four-profile solution); the absence of conceptually distinct new subgroups in the four-profile solution, which subdivides the Medium class into two severity-adjacent classes rather than revealing a new, qualitatively meaningful subgroup; and interpretability. In addition, the class proportions in the three-profile model were relatively balanced (64.60%, 23.92%, and 11.48%), with the smallest class comprising 11.48% of the sample—well above the recommended minimum threshold of 5%. Accordingly, the three-profile solution is presented as a substantive choice rather than as the statistically best-fitting model, and the four-profile solution was examined as a robustness check (Section 3.6 and Supplementary Table 1).

Based on the scoring characteristics of each class across the four dimensions of AI anxiety in the retained three-profile solution (see Figure 1), the profiles were labeled as follows. Class 1 (C1) was designated as the “Medium AI Anxiety Group,” comprising 321 students (65.78%). Members of this profile exhibited a relatively stable anxiety pattern and represented the most prevalent class among medical imaging students. Class 2 (C2) was designated as the “High AI Anxiety Group,” comprising 111 students (22.75%). Members of this profile scored particularly high on the Job Replacement anxiety and Sociotechnical Blindness anxiety dimensions, reflecting deep-seated concerns about AI-driven occupational displacement and socioethical implications. Class 3 (C3) was designated as the “Low AI Anxiety Group,” comprising 56 students (11.48%). Members of this profile scored low across all four dimensions, indicating a relatively calm and positive psychological disposition toward the development of AI technology.

**Figure 1:**
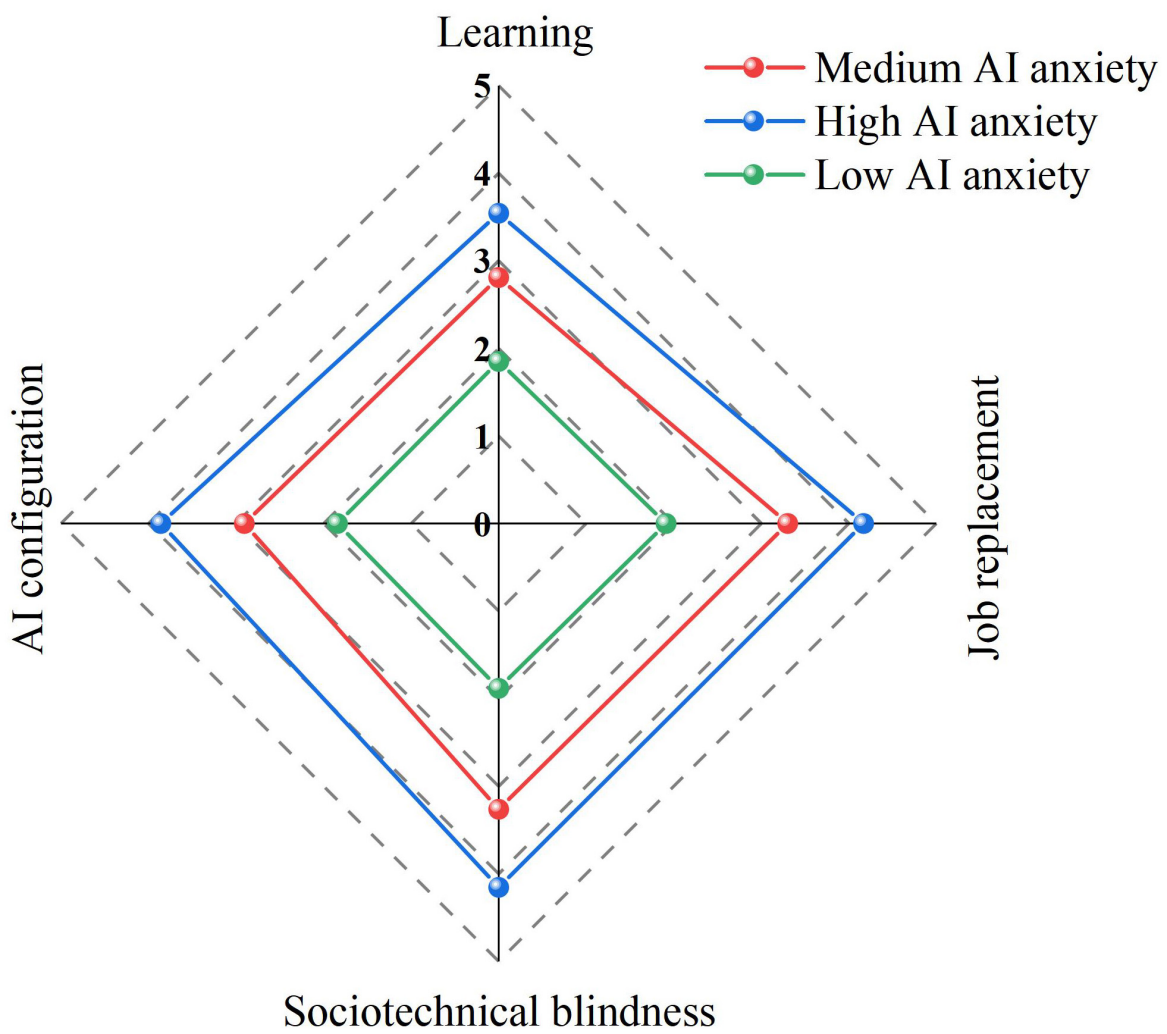
Each class across the four dimensions of AI anxiety in the optimal 3-profile model.

### 3.4 Profile characteristics (indicator means)

The three profiles differed primarily in severity (level) rather than shape. The profile-specific indicator means, standard errors, and 95% confidence intervals are reported in Table 5. The correlations among the profile indicator-mean vectors were *r* = 0.956 (Medium vs. High), *r* = 0.916 (Medium vs. Low), and *r* = 0.788 (High vs. Low), indicating broadly parallel profiles. The three profiles were broadly parallel in their internal ordering, with Job Replacement and Sociotechnical Blindness most elevated, followed by AI Configuration and Learning; the only exception was a minor reversal in the Low profile, where Learning (1.850) was marginally higher than AI Configuration (1.841). Class 1 (*n* = 321, 65.78%) was labeled the Medium AI anxiety profile; Class 2 (*n* = 111, 22.75%) the High AI anxiety profile, with particularly pronounced Job Replacement and Sociotechnical Blindness concerns; and Class 3 (*n* = 56, 11.48%) the Low AI anxiety profile. The average latent-class posterior probabilities were 0.939, 0.901, and 0.946, respectively.

**Table 5:** The profile-specific indicator means, standard errors, and 95% confidence intervals.

Profile (*n*, %)	Learning *M* (SE) (95% CI)	Job replacement *M* (SE) (95% CI)	Sociotechnical blindness *M* (SE) (95% CI)	AI configuration *M* (SE) (95% CI)
Medium (321, 65.78%)	2.808 (0.043) (2.72, 2.89)	3.301 (0.071) (3.16, 3.44)	3.263 (0.067) (3.13, 3.39)	2.905 (0.055) (2.80, 3.01)
High (111, 22.75%)	3.543 (0.147) (3.25, 3.83)	4.168 (0.084) (4.00, 4.33)	4.156 (0.094) (3.97, 4.34)	3.860 (0.168) (3.53, 4.19)
Low (56, 11.48%)	1.850 (0.093) (1.67, 2.03)	1.912 (0.099) (1.72, 2.11)	1.883 (0.135) (1.62, 2.15)	1.841 (0.125) (1.60, 2.09)

### 3.5 Differential analysis across the retained latent profiles

To examine distributional differences in demographic and academic characteristics across the different AI anxiety latent profiles, chi-square tests and one-way analyses of variance (ANOVA) were conducted. The results are summarized in Table 6.

**Table 6:** Differential analysis across the optimal latent profiles.

Variables	Items	Medium AI anxiety	High AI anxiety	Low AI anxiety	χ^2^/F	*p*
Gender	Male	150	50	33	3.264	0.195
	Female	171	61	23		
Grade	Freshman year	129	39	16	7.188	0.516
	Sophomore Year	88	28	15		
	Junior Year	55	26	15		
	Senior year	21	10	6		
	Fifth year of university	28	8	4		
Educational system	Four-year program	144	54	31	2.281	0.320
	Five-year program	177	57	25		
Major	Medical imaging technology	137	37	21	3.162	0.206
	Medical imaging diagnosis	184	74	35		
Only child	No.	191	56	20	11.942	0.003
	Yes.	130	55	36		
Internship or internship experience	No.	200	67	27	3.954	0.138
	Yes.	121	44	29		
Frequency of AI use	1~2 times	152	47	24	6.895	0.331
	3~5 times	95	35	14		
	6 times and above	60	28	15		
	Unused	14	1	3		
Age	M ± SD	21.15 ± 3.290	20.40 ± 2.024	21.25 ± 3.380	2.709	0.068
Emotional flexibility	M ± SD	3.316 ± 0.431	3.753 ± 0.589	3.937 ± 1.001	45.299	<0.001
Future employment anxiety	M ± SD	3.291 ± 0.485	4.039 ± 0.463	2.337 ± 0.726	210.954	<0.001

No statistically significant differences were observed across the three latent profiles for gender (χ^2^ = 3.264, *p* = 0.195), year of study (χ^2^ = 7.188, *p* = 0.516), program duration (χ^2^ = 2.281, *p* = 0.320), medical imaging major (χ^2^ = 3.162, *p* = 0.206), internship/clinical placement experience (χ^2^ = 3.954, *p* = 0.138), or frequency of AI use (χ^2^ = 6.895, *p* = 0.331). Notably, only-child status exhibited a statistically significant difference across the three AI anxiety latent profiles (χ^2^ = 11.942, *p* = 0.003).

The one-way ANOVA results revealed highly significant differences across the three AI anxiety latent profiles in emotional flexibility (*F* = 45.299, *p* < 0.001). Although the Medium AI Anxiety Group exhibited a lower overall level of AI anxiety than the High AI Anxiety Group, its emotional flexibility was the lowest among the three groups. This finding suggests that students in the Medium AI Anxiety Group may tend to adopt relatively rigid emotional coping strategies when confronted with the uncertainty introduced by AI, and that their medium level of anxiety may reflect limitations in emotion regulation strategies rather than genuine psychological adaptation. In contrast, the Low AI Anxiety Group demonstrated the highest level of emotional flexibility, indicating that individuals with greater emotional flexibility are more effective at regulating and managing the psychological stress arising from AI-driven technological change, thereby maintaining lower levels of AI anxiety.

Highly significant differences were also observed across the three latent profiles in future employment anxiety (*F* = 210.954, *p* < 0.001). The High AI Anxiety Group (C2) exhibited significantly higher levels of future employment anxiety than the other two groups (*M* = 4.039, SD = 0.463), indicating that students in this group harbored particularly intense concerns about their future career prospects. The Medium AI Anxiety Group (C1) displayed a moderately elevated level of future employment anxiety (*M* = 3.291, SD = 0.485). The Low AI Anxiety Group (C3) reported significantly lower future employment anxiety than the other two groups (*M* = 2.337, SD = 0.726), reflecting a relatively optimistic and composed outlook toward future career development.

BCH distal-outcome analysis (Table 7) showed significant overall differences across the three profiles in emotional flexibility (χ^2^ = 68.798, *p* < 0.001) and future employment anxiety (χ^2^ = 342.361, *p* < 0.001). Emotional flexibility was highest in the Low profile (*M* = 3.975, *SE* = 0.141), lowest in the Medium profile (*M* = 3.282, *SE* = 0.026), and intermediate in the High profile (*M* = 3.805, *SE* = 0.063); the Medium profile was significantly lower than both the High (*p* < 0.001) and Low (*p* < 0.001) profiles, whereas the High and Low profiles did not differ significantly (*p* = 0.271). Future employment anxiety increased monotonically from the Low (*M* = 2.285, *SE* = 0.102) through the Medium (*M* = 3.255, *SE* = 0.030) to the High (*M* = 4.126, *SE* = 0.050) profile (all pairwise *p* < 0.001).

**Table 7:** BCH distal-outcome means (classification-error-adjusted) across the three latent profiles.

Distal outcome	Medium *M* (SE)	High *M* (SE)	Low *M* (SE)	Overall χ^2^ (*p*)	Significant pairwise contrasts
Emotional flexibility	3.282 (0.026)	3.805 (0.063)	3.975 (0.141)	68.798 (<0.001)	Medium <High (*p* < 0.001); Medium <Low (*p* < 0.001); High vs. Low (*p* = 0.271)
Future employment anxiety	3.255 (0.030)	4.126 (0.050)	2.285 (0.102)	342.361 (<0.001)	All pairwise contrasts *p* < 0.001

Because Levene's test indicated heterogeneous variances (emotional flexibility *W* = 70.682, *p* < 0.001; future employment anxiety *W* = 11.759, *p* < 0.001), Welch's ANOVA and Games–Howell comparisons were used: emotional flexibility Welch *F*_(2, 111.7)_ = 33.875, *p* < 0.001; future employment anxiety Welch F _(2, 123.6)_ = 171.626, *p* < 0.001. Games–Howell contrasts and Cohen's d values are reported in Table 8. The univariate R3STEP analysis (only-child status as the sole covariate) showed that only-child status was associated with membership in the Low (vs. Medium) profile in the unadjusted R3STEP analysis [*B* = 1.068, *SE* = 0.330, OR = 2.909, 95% CI (1.524, 5.556), *p* = 0.001], but this association was attenuated to non-significance in the multivariate, classification-error-adjusted model (Low vs. Medium: *p* = 0.287; High vs. Medium: *p* = 0.356), indicating that the only-child effect is substantially accounted for by emotional flexibility and future employment anxiety.

**Table 8:** Welch's ANOVA and Games–Howell *post hoc* comparisons with effect sizes.

Variable	Welch F	*p*	Games–Howell contrasts	Cohen's d
Emotional flexibility	*F*_(2, 111.7)_ = 33.875	<0.001	Medium <High (*p* < 0.001); Medium <Low (*p* < 0.001); High vs. Low (*p* = 0.420)	−0.919; −1.122; −0.244
Future employment anxiety	*F*_(2, 123.6)_ = 171.626	<0.001	Medium <High (*p* < 0.001); Medium > Low (*p* < 0.001); High > Low (*p* < 0.001)	−1.560; 1.809; 3.014

### 3.6 Independent predictors of latent profile membership (classification-error-adjusted)

To identify which correlates independently characterized profile membership while accounting for classification uncertainty, emotional flexibility, future employment anxiety, and only-child status were entered simultaneously as covariates in an R3STEP multinomial regression, with the Medium profile (the modal class) as the reference category. Emotional flexibility and future employment anxiety are conceptualized here as distal correlates that differentiate the profiles (their classification-error-adjusted means are compared via BCH in Section 3.4); the R3STEP analysis is a purpose-specific test of their independent predictive value, and no variable serves as both outcome and covariate within a single model. The results are reported in Table 9.

**Table 9:** Independent predictors of latent profile membership (classification-error-adjusted).

Contrast (vs. Medium)	Predictor	*B*	SE	OR	95% CI	*p*
High	Emotional flexibility	1.317	0.476	3.733	[1.468, 9.487]	0.006
High	Future employment anxiety	3.804	0.575	44.902	[14.54, 138.5]	<0.001
High	Only child	0.350	0.379	1.419	[0.675, 2.983]	0.356
Low	Emotional flexibility	0.490	0.745	1.632	[0.379, 7.030]	0.511
Low	Future employment anxiety	−3.337	1.299	0.036	[0.003, 0.453]	0.010
Low	Only child	0.649	0.610	1.913	[0.579, 6.325]	0.287

Reference category = Medium profile; OR, odds ratio; CI, confidence interval; p-values are Wald tests of the logit coefficients (coefficient scale).

#### 3.6.1 High versus medium

Future employment anxiety significantly predicted membership in the High (vs. Medium) profile [*B* = 3.804, SE = 0.575, *p* < 0.001, OR = 44.902, 95% CI (14.54, 138.5)]; each one-unit increase in future employment anxiety substantially increased the odds of belonging to the High relative to the Medium profile. Emotional flexibility also significantly distinguished the two profiles [*B* = 1.317, *SE* = 0.476, *p* = 0.006, OR = 3.733, 95% CI (1.468, 9.487)]. Only-child status was not significant (*B* = 0.350, SE = 0.379, *p* = 0.356, OR = 1.419).

#### 3.6.2 Low versus medium

Future employment anxiety was again a significant predictor, such that lower future employment anxiety was associated with membership in the Low (vs. Medium) profile [B = −3.337, SE = 1.299, *p* = 0.010, OR = 0.036, 95% CI (0.003, 0.453)]; the wide confidence interval reflects the small size of the Low class). Emotional flexibility did not significantly distinguish the Low from the Medium profile (*B* = 0.490, SE = 0.745, *p* = 0.511, OR = 1.632), and only-child status was not significant (*B* = 0.649, SE = 0.610, *p* = 0.287, OR = 1.913).

In summary, after correcting for classification uncertainty, future employment anxiety was the most robust independent predictor distinguishing all three profiles, whereas emotional flexibility distinguished the High from the Medium profile but not the Low from the Medium profile. Variance inflation factors for the three covariates were all below conventional thresholds (emotional flexibility = 1.024; future employment anxiety = 1.008; only-child = 1.031), indicating that multicollinearity among predictors was not problematic.

### 3.7 Robustness check and sensitivity analysis

#### 3.7.1 Four-profile solution

The four-profile solution subdivided the Medium class into two severity-adjacent classes (medium-low: Job Replacement = 3.050; medium-high: Job Replacement = 3.777) that did not differ in shape; it was therefore not retained as the primary model. The four-profile indicator means (with standard errors) are reported in Supplementary Table 1. Under the four-profile solution, the distal-outcome conclusions were substantively identical to those of the three-profile solution: future employment anxiety increased monotonically across the four severity-ordered classes (BCH means 2.272, 3.035, 3.726, and 4.251; χ^2^ = 453.228, *p* < 0.001), and emotional flexibility again showed the non-linear pattern with the medium classes lowest (BCH means 4.218, 3.211, 3.481, and 3.949; χ^2^ = 92.688, *p* < 0.001). This confirms that the principal findings are robust to the three- vs. four-profile choice.

Targeted overlap check (reduced future-employment-anxiety score). Excluding the four Career Replaceability items yielded a reduced future-employment-anxiety score that correlated r = 0.991 with the full score (98.2% shared variance), so this check is best interpreted as removing only the most directly overlapping component rather than as a test of discriminant validity. With the reduced score, future employment anxiety still differentiated the profiles (BCH: Medium = 3.253, High = 4.105, Low = 2.324; overall χ^2^ = 332.389, *p* < 0.001; all pairwise *p* < 0.001), and it remained a significant independent predictor in the classification-error-adjusted model (High vs. Medium: B = 3.920, SE = 0.663, *p* < 0.001; Low vs. Medium: *B* = −2.958, SE = 1.035, *p* = 0.004). Thus, the association between future employment anxiety and AI-anxiety profile membership persisted after excluding the most directly overlapping component.

## 4 Discussion

### 4.1 Latent profile analysis of AI anxiety

The present study revealed that AI anxiety among medical imaging students is best described by three severity-ordered profiles—the Medium (65.78%), High (22.75%), and Low (11.48%) AI anxiety profiles—that differ primarily in overall level rather than in distinct shape. Across all three profiles, Job Replacement and Sociotechnical Blindness concerns were the most elevated dimensions, suggesting that the core of AI anxiety in this population is socio-occupational rather than technical. Although the profiles are quantitatively differentiated, the person-centered approach retains its value by identifying a distinct high-severity subgroup requiring intensive intervention and by revealing a non-linear association between AI anxiety and emotional flexibility that a variable-centered analysis could not capture.

The Medium AI Anxiety Group, as the largest class, constitutes the predominant segment of the medical imaging student population. A Medium level of anxiety arousal facilitates the maintenance of cognitive sensitivity and behavioral readiness in response to environmental change (Easterbrook, 1959). For the majority of medical imaging students, awareness of the increasing penetration of AI technology into their professional domain has generated a certain degree of concern and apprehension (Ampofo et al., 2023); however, this concern has not yet reached a level that significantly disrupts psychological functioning. This finding suggests that, against the backdrop of rapid AI advancement, most medical imaging students—while cognizant of the challenges that technological transformation may entail—have maintained a relatively adaptive psychological state overall.

Although the High AI Anxiety Group accounted for only 22.75% of the total sample, members of this group scored particularly high on the Job Replacement Anxiety and Sociotechnical Blindness Anxiety dimensions, indicating a more pronounced activation of anxiety along specific dimensions. According to the technology threat perception theory (Liang and Xue, 2009), when individuals appraise AI technology as a direct threat to their professional roles, the resulting job replacement anxiety response is most intense (Chen and Ke, 2025). The high diagnostic accuracy of deep learning algorithms in medical imaging has been substantiated by a substantial body of empirical research (Aggarwal et al., 2021), and such visible evidence of replaceability may activate existential career anxiety among medical imaging students more readily than among students in other medical specialties. The prominence of Sociotechnical Blindness Anxiety reflects students' concerns about broader socioethical implications of AI technology (Babanoglu et al., 2025), including deep-seated unease regarding AI decision-making transparency, algorithmic bias, and the attribution of medical liability. The identification of this compound anxiety pattern suggests that this group requires comprehensive psychological support encompassing not only technical competency enhancement but also professional identity reconstruction and ethical cognition clarification.

The Low AI Anxiety Group comprised the smallest proportion of the sample, indicating that even within a professional field highly associated with AI technology, a subset of students maintains a relatively positive and composed psychological orientation toward technological change. This psychological disposition may arise from the synergistic interplay of multiple factors, including higher technological self-efficacy, a rational understanding of AI as an assistive rather than a substitutive tool, and more flexible emotion regulation capabilities.

### 4.2 Analysis of influencing factors

#### 4.2.1 Emotional flexibility

Emotional flexibility showed a non-linear association with AI anxiety profiles: it was lowest in the Medium profile and highest in the Low profile, with the High profile intermediate. One possible—and tentative—interpretation is that students in the Medium profile may sustain an ostensibly medium anxiety level through relatively rigid emotion-regulation strategies (e.g., avoidance or suppression of AI-related concerns), such that their medium anxiety reflects limited regulatory flexibility rather than genuine adaptation; this mechanistic account, however, was not directly measured and should be treated as speculative. The finding that the High and Low profiles did not differ in emotional flexibility (*p* = 0.271) further underscores the complexity of this association. In the classification-error-adjusted (R3STEP) analysis, emotional flexibility distinguished the High from the Medium profile but not the Low from the Medium profile, suggesting that any protective effect of emotional flexibility may be partially indirect through reduced future employment anxiety.

#### 4.2.2 Future employment anxiety

Future employment anxiety was the most robust independent predictor distinguishing the profiles, increasing monotonically from the Low through the Medium to the High profile. Consistent with a cognitive–emotional spillover account, students' AI-related concerns may generalize to broader career uncertainty. However, because the design is cross-sectional, the direction of this association cannot be established, and it is equally plausible that pre-existing career anxiety heightens AI-specific concerns. These processes could reinforce one another over time, a possibility that requires longitudinal testing to establish. More importantly, the core concern underlying AI anxiety among medical imaging students may not be the complexity or learning difficulty of the technology itself, but rather the existential career threat triggered by technological transformation. When individuals perceive that the career trajectory upon which they have constructed their life meaning faces fundamental challenges, the anxiety thus engendered possesses a deeply existential quality whose intensity far surpasses surface-level concerns about specific difficulties in adapting to new technologies. A sensitivity analysis confirmed that this association was not an artifact of the shared 'replaceability' content between the two instruments, as it persisted after the Career Replaceability items were removed.

With the exception of only-child status, demographic and academic variables did not differentiate the profiles. Only-child status was associated with a higher likelihood of belonging to the Low (vs. Medium) profile in the unadjusted R3STEP analysis [OR = 2.909, 95% CI (1.524, 5.556), *p* = 0.001], which may reflect the more abundant family resources typical of single-child families. However, this association was attenuated to non-significance in the multivariate, classification-error-adjusted model (Low vs. Medium: *p* = 0.287; High vs. Medium: *p* = 0.356), indicating that the only-child effect is substantially accounted for by emotional flexibility and future employment anxiety. AI-use frequency did not differentiate the profiles, possibly because familiarity-based anxiety reduction and threat-appraisal-based anxiety amplification operate in opposing directions and cancel out at the group level.

### 4.3 Practical implications

The findings of the present study suggest that educators should adopt a differentiated intervention approach based on profile classification. For the Medium AI Anxiety Group, which constitutes the largest proportion and is characterized by the lowest level of emotional flexibility, psychological flexibility training modules grounded in Acceptance and Commitment Therapy could be systematically embedded within the curriculum to help students develop psychological acceptance of AI-related uncertainty and cognitive defusion skills. For the High AI Anxiety Group, intervention efforts should focus on career cognition restructuring and AI technology empowerment. This may be achieved through offering practice-oriented courses centered on human-AI collaboration, organizing dialogue and exchange activities with AI industry practitioners, and providing specialized career psychological counseling services to help students construct positive cognitive frameworks regarding their professional roles in the AI era and substantively reduce their future employment anxiety.

The necessity of integrating AI technology literacy education into the core curriculum of medical imaging programs is further supported by the findings of this study. Helping students systematically understand the positioning of AI within the imaging field—specifically, AI's role as an assistive tool that augments rather than replaces professional judgment—may help correct catastrophizing cognitions about AI replacing human workers at their source, thereby alleviating anxiety responses triggered by perceived career threats.

The AI anxiety profile structure identified in this study can provide a precision-oriented screening framework for university mental health service systems. It is recommended that departments associated with medical imaging regularly conduct AI anxiety assessments, with particular attention to students who may belong to the High AI Anxiety profile, and develop individualized psychological support plans tailored to their levels of future employment anxiety and emotional flexibility characteristics.

### 4.4 Limitations and future research directions

Several limitations warrant consideration. First, the cross-sectional design precludes causal inference; longitudinal and cross-lagged designs are needed to establish the direction of the associations. Second, convenience sampling from a medical colleges in Sichuan Province limits geographic and institutional generalizability. Third, all measures were self-report and are susceptible to social-desirability and recall bias; although Harman's single-factor test suggested common method bias was not severe, this test is only a diagnostic and does not statistically control for method effects, so future studies should incorporate multi-informant or objective indicators. Fourth, the LPA assumed a class-invariant diagonal covariance structure; alternative specifications (e.g., class-varying variances) could be explored in larger samples. Fifth, the Emotional Flexibility Scale was originally developed among adolescent earthquake survivors; although its items concern general emotion-regulation flexibility and it showed good reliability in our sample, its suitability for general undergraduate populations warrants further validation. Sixth, the mechanistic interpretations (e.g., avoidance, suppression, “defensive equilibrium”) concern processes not directly measured and remain tentative. Seventh, although the three-profile solution was retained for parsimony, classification precision, and interpretability, the four-profile solution received stronger statistical support (lower information criteria and a significant LMR-LRT), and future studies with larger samples should adjudicate between these solutions. Finally, a confirmatory factor analysis indicated substantial shared variance between AI anxiety and future employment anxiety (latent *r* = 0.792), and the Fornell–Larcker criterion for discriminant validity was not met; although a two-factor model fitted better than a one-factor model, the two constructs are substantively adjacent (particularly Job Replacement ↔ Career Replaceability), and our targeted overlap check (excluding Career Replaceability) should not be interpreted as establishing full discriminant validity.

## 5 Conclusion

The present study used latent profile analysis to identify three severity-ordered AI anxiety profiles among medical imaging students: Medium, High, and Low. Emotional flexibility showed a non-linear association with these profiles, with the Medium profile exhibiting the lowest emotional flexibility. In the classification-error-adjusted analysis, future employment anxiety was the most robust independent predictor distinguishing the profiles and was strongly associated with AI anxiety, an association that persisted after excluding the conceptually overlapping Career Replaceability items. These findings support profile-based differentiated psychological interventions and the systematic integration of AI literacy and career planning into medical imaging curricula.

## Data Availability

The raw data supporting the conclusions of this article will be made available by the authors, without undue reservation.
