# Supplementary material for: Latent profile analysis of AI anxiety among medical imaging students: a cross-sectional study based on emotional flexibility and future employment anxiety
Source: Front Psychol. 2026 Oct 20;17:1882645. doi: 10.3389/fpsyg.2026.1882645 (PMC13727622; doi:10.3389/fpsyg.2026.1882645)
Supplement: Supplementary file 1 [file Table_1.docx]

# Latent Profile Analysis of AI Anxiety Among Medical Imaging Students: A Cross-Sectional Study Based on Emotional Flexibility and Future Employment Anxiety

| Profile (n, %) | Learning | Job Replacement | Sociotechnical Blindness | AI Configuration |
| --- | --- | --- | --- | --- |
| Low (9.43%) | 1.795 (0.102) | 1.818 (0.109) | 1.726 (0.140) | 1.735 (0.144) |
| Moderate-low (42.42%) | 2.741 (0.047) | 3.050 (0.055) | 2.991 (0.048) | 2.803 (0.050) |
| Moderate-high (36.89%) | 3.016 (0.059) | 3.777 (0.047) | 3.769 (0.053) | 3.216 (0.089) |
| High (11.27%) | 3.814 (0.122) | 4.378 (0.080) | 4.433 (0.099) | 4.199 (0.090) |
